# Attitudes of the Ecuadorian University Community Toward Genetically Modified Organisms

**DOI:** 10.3389/fbioe.2021.801891

**Published:** 2022-02-18

**Authors:** Carlos Román Collazo, Karen Chacha Guerrero, Tatiana Loja Mejia, Diego Andrade Campoverde, Yenima Hernández Rodriguez

**Affiliations:** ^1^ Faculty of Biochemistry and Pharmacy, Catholic University of Cuenca, Cuenca, Ecuador; ^2^ Faculty of Clinical Psychology, Universidad Católica de Cuenca, Cuenca, Ecuador

**Keywords:** attitudes, genetically modified organisms, biotechnology, transgenic, genetic engineering

## Abstract

**Introduction:** The acceptance of genetically modified organisms (GMOs) by the civilian population in Ecuador is a controversial issue, where beliefs and practices are determinant. In Ecuador, the use of GMOs for research or productive purposes has been banned since 2008; however, the current position of the population toward this technology is unknown.

**Objective:** The aim of the study was to explain the attitude toward GMOs in the Ecuadorian university population based on sociodemographic variables, knowledge, beliefs, practices, and bioethical approach.

**Methods:** A validated survey was applied to 719 students and teachers of the Catholic University of Cuenca through Google Forms. The collected data were processed using SPSS 23.0 software. Multivariate and linear regression analyses were used to explain the attitude toward GMOs based on the variables studied.

**Results:** Partial approval of GMO use is research-oriented, with a rejection toward food. The linear regression model explained 65% of the variance of attitude toward GMOs from the beliefs, practices, knowledge, and bioethical approach variables. The sociodemographic variables were completely excluded from the model due to the absence of statistical significance.

**Conclusions:** The incipient acceptance of GMOs in the academic sector corroborates a transformation in the thinking of Ecuadorian civil society. Considerations on the use of GMOs are supported by a bioethical approach that leans toward a pragmatic utilitarianism based on the immediate or mediate benefits of the technology.

## Introduction

The use of genetically modified organisms (GMOs) in research, health, and food is a reality since the end of the 20th century (Manoj and Ratwan, 2018) (Robinson AW and Rajakaruna, 2016). After almost 40 years of its establishment as a technology, its acceptance and use are spreading to countries in several continents, mainly America (Paull and Hennig, 2019).

Even when there is unobjectionable evidence of the socioeconomic advantages of GMOs (Smyth et al., 2015); some regions of the planet are reluctant to adopt them as part of technological or productive systems (Martin et al., 2017). In Latin America, countries such as Venezuela, Peru, and Ecuador continue to object to the implementation of GMO technology under the bioethical principle of precaution, hindering its development at regional and local levels (Gatica-Arias, 2020).

In Ecuador, the constitution and other legal figures limit GMO research and production (Gudynas, 2017), even though consulting entities have suggested its application at the local level (Trigo et al., 2002). It is also paradoxical in this prohibitive context, the use of GMO products by the population in high diversity and magnitude (Galeas, Yépez, and Lascano, 2016).

In 2008, a massive consultation about GMOs was held within the framework of the new Ecuadorian constitution. At this time, Ecuador was considered free of “transgenic organisms” and “risky biotechnologies” (Bravo 2017). Although there are investigations on the acceptance of GMOs in the Ecuadorian population, they are restrained to food consumption. There is no reference to the current perception of the Ecuadorian population toward GMOs in research or pharmaceutical applications. It is also unknown what variables may be associated with the position of the Ecuadorian population toward GMOs. The objective of the research was to explain the attitudes toward GMOs in the Ecuadorian university population based on sociodemographic variables, beliefs, practices, and bioethical approach.

## Materials and Methods

### Research Design

The research followed a non-experimental, observational, cross-sectional, and explanatory design. The study population was the university community of the Catholic University of Cuenca (UCACUE), Ecuador, during the period March–August 2020. The total population was 14,482 teachers and students. The sampling was non-probabilistic, reaching the total study population. The inclusion criterion was to be a member of UCACUE during the research period. The exclusion criterion was to perform service or administrative functions at UCACUE. The sample was 729 members of the educational community.

### Survey

A survey on attitudes toward GMOs was elaborated according to Pardo and collaborators (Pardo, Midden, and Miller 2002) (Annex 1). The survey was applied online using the Google Forms platform, which was administered through the UCACUE email management system. The survey was available online from November 2019 to August 2020. The survey was previously validated by piloting with reliability by Cronbach’s alpha (*α* = 0.81) and content validity by V Aiken (0.82–0.97). The dimension practices with GMOs (6 items; *α* = 0.721; V Aiken = 0.84–0.96), attitude toward GMOs (6 items; *α* = 0.862; V Aiken = 0.89–0.97), beliefs regarding GMOs (16 items; *α* = 0.883; V Aiken = 0.89–0.97), and the bioethical approach (5 items; *α* = 0.796; V Aiken = 0.81–0.94) were measured on a Likert scale (1, strongly disagree, to 5, strongly agree). In addition, GMO knowledge (9 items; *α* = 0.869; V Aiken_0.86–0.97) was assessed on a scale of 0–9. Sociodemographic variables such as age, sex, place of residence, religion, educational level, academic training, family economic income, self-perceived GMO knowledge, and food expenditures were explored. The survey was self-administered with an average response time of 12 min.

### Statistical Processing

The data were stored in an electronic database and processed using IBM SPSS 23.0 statistical software. Frequency analysis, measures of central tendency and position (mean, confidence intervals, percentiles), and dispersion (standard deviation, range) were used. Differences between groups were established using the Wilcoxon test for comparison of means for different groups. The effect of the independent variables was done by multivariate regression analysis and subsequently by linear regression. The assumptions of the model such as the absence of collinearity were analyzed by graphs, the Durbin Watson coefficient, and correlation between the independent variables. The significance level of all tests was less than or equal to 0.050.

### Ethical Aspects

The research complied with the ethics of research with human subjects using informed consent and the voluntary approval of the participants. Prior informed consent was obtained from all those involved. The research objectives were presented in writing, highlighting the importance of the research. Anonymity and the willingness of respondents and interviewees to disclose information were respected.

## Results

### Sociodemographic Characteristics of the Sample

The average age of the sample was 36.92 +− 11.61 (95% CI, 36.07–37.76) years. There was similarity in the proportions of men (48.6%) and women (51.4%) (X^2^ = 0.605; *p* = 0.437), with urban residence being the majority (83.7%) over rural residence (16.3%) (X^2^ = 330.701; *p* = 0.000). The educational level of the majority was in the Master’s–Doctorate category (51.3%), decreasing for higher basic level (28.0%) and university level (20.7%) (X^2^ = 111.712; *p* = 0.000).

**FIGURE 1:**
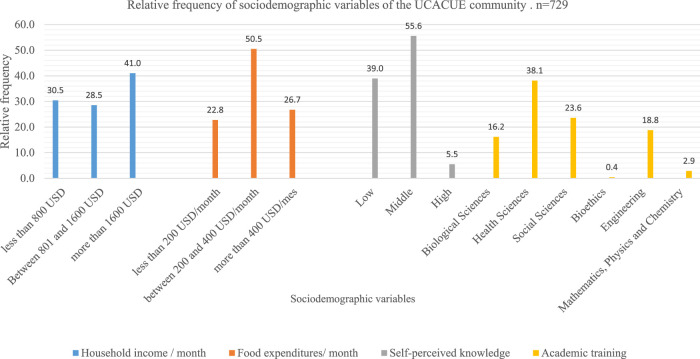
Sociodemographic characteristics of the UCACUE community, March–August 2020, Ecuador.

Figure 1 shows the relative frequency of the variables household income, food expenditures, self-perceived knowledge about GMOs, and academic training. The most frequent family income was higher than 1,600 USD (X^2^ = 111.712; *p* = 0.000). Expenditures on food were centered between 200 and 400 USD (X^2^ = 98.181; *p* = 0.000). The predominant self-perceived knowledge corresponded to the medium category (X^2^ = 284.502; *p* = 0.000). The predominant training area was health sciences, followed by social sciences. The low level of training in bioethics of the population is highlighted (X^2^ = 224.126; *p* = 0.000).

The majority of the sample is religiously Catholic (80.7%). The rest of the categories such as Protestant (4.9%), Muslim (0.5%), Afro-Ecuadorian (0.3%), and others (3.8%) showed lower frequencies (X^2^ = 2,174.410; *p* = 0.000). A total of 9.7% did not adhere to any religion.

### Attitude Toward GMOs

Table 1 shows the descriptive statistics of the attitude, beliefs, practices, bioethics approach, and knowledge variables on GMOs. The average knowledge of the sample is located in the second quartile of the sampling distribution, suggesting a low level of familiarity with GMOs. Beliefs showed mean values close to the middle of the measurement scale. Practices showed a mean value below the middle of the scale, indicating less activity of individuals with GMOs in their daily lives. The bioethical approach also had a medium value, suggesting a deficit of bioethical thinking in the educational community. Finally, attitude toward GMOs, similar to the dimensions, showed a medium value in its behavior.

**TABLE 1:** Descriptive statistic of the attitude, beliefs, practices, and knowledge on GMO variables in UCACUE, 2020.

Variable	X +− SD	CI 95%	Min-Max	Instrument scale
Attitude toward GMO	3.03 +− 1.01	2.96–3.11	1–5	1–5
Beliefs about GMO	3.33 +− 0.70	3.28–3.38	1–5	1–5
Practices with GMO	2.54 +− 0.80	2.48–2.60	1–5	1–5
Knowledge about OGM	1.95 +− 1.66	1.81–2.05	0–8	0–9
Bioethical approach	2.98 +− 0.94	2.92–3.06	1–5	1–5

X +− SD-mean +− standard deviation.

CI, confidence interval.

Min, minimum.

Max, maximum.

Table 2 shows the attitude toward GMOs according to the items that make up the dimension—the items with the lowest scores corresponded to the use of GMOs as food in humans or livestock. The highest acceptance corresponded to the use of GMOs as a means of research. There was a medium consensus on the use of GMOs for environmental protection under appropriate biosafety standards.

**TABLE 2:** Items that make up the attitude toward GMOs dimension.

Items that make up the attitude toward GMO dimension	Mean	Standard deviation	Mean’s comparison (Wilcoxon’s test)
A. I approve of the use of GMOs technology in the country under strict biosafety regulations	3.17	1.21	B > A
B. I am in favor of the use of GMOs in scientific research	3.39	1.20	Z = −5,638 *p* = 0.000
C. I approve GMOs for human consumption	2.72	1.17	A = F
D. I approve GMOs for feeding farmed animals	2.71	1.18	Z = −0.309 *p* = 0.758
E. I approve GMOs to produce medicines for humans and animals	3.06	1.18	A > C
F. I approve the use of GMOs for the care of the environment	3.16	1.26	Z = −10,822 *p* = 0.000
			C = D
			Z = −0.570 *p* = 0.568

The modeling of attitude toward GMOs showed the properties of the model and the explanatory variables (Table 3). The proposed model was statistically significant in explaining the attitude toward GMOs of the UCACUE educational community. The variables adjusted to the model were beliefs, practices, knowledge, and bioethical approach toward GMOs by showing statistical significance. The variables entered showed a Durbin–Watson coefficient of 1.95, with tolerance and VIF adequate, ensuring the absence of collinearity and correlation between the explanatory variables. The rest of the variables was discarded because their inclusion in the model was not significant. The calculated coefficient of determination explains 65% of the attitude with a respective contribution of beliefs (43.4%), practices (14.7%), bioethical approach (10.5%), and knowledge (5%). Therefore, the modeling equation responds to the straight line shown:

Attitude towards GMO= − 0.674+0.434(Beliefs)+0.147(Practices)+0.050(Knowledge)+0.105(Bioethical approach) .

**TABLE 3:** Summary measures of the linear regression model.^a^ ^,^ ^b^

R	R^2^	Adjusted R^2^	Standard error R2	Change statistics					Durbin–Watson	ANOVA
				R^2^ change	F change	df1	df2	Sig. F change		
0.807	0.652	0.647	0.600	0.652	149.444	10	719	0.000	1.951	F = 149.44 p = 0.000

Model				Unstandardized coefficients		Standardized coefficients	t	Sig	95.0% confidence interval B	
				B	Standard error	Beta			Lower limit	Upper limit
(Constant)				−0.697	0.165		−4.235	0.000	−1,021	−,374
Sex				0.078	0.046	0.039	1.709	0.088	−0.012	0.168
Place of residence				0.069	0.061	0.025	1.118	0.264	−,052	0.189
GMO beliefs				0.432	0.043	0.300	9.998	0.000	0.347	0.516
Practices with GMOs				0.147	0.033	0.117	4.507	0.000	0.083	0.211
GMO knowledge				0.050	0.014	0.082	3.460	0.001	0.021	0.078
Bioethical approach toward GMO				0.106	0.006	0.495	16.867	0.000	0.094	0.118
Religion				0.020	0.014	0.033	1.472	0.141	−0.007	0.048
Food expenditures (USD)				−0.047	0.038	−0.032	−1.217	0.224	−0.122	0.029
Household income (USD)				0.051	0.033	0.042	1.567	0.118	−0.013	0.115
Educational level				0.005	0.038	0.004	0.136	0.892	−0.069	0.080
Academic training				0.011	0.016	0.015	0.663	0.508	−0.21	0.42

a Predictors: (constant), sex, place of residence, GMO beliefs, practices with GMOs, GMO knowledge, bioethical approach toward GMO, religion, food expenditures (USD), household income (USD), educational level, and academic training.

b Dependent variable: attitude toward GMOs.

## Discussion

Public attitudes towards biotechnology have been explored since the emergence of GMOs. The introduction of GMOs into human life has been marked by a dichotomy: acceptance or rejection. Their varied applications lead to a different position depending on the usefulness of the GMO, revealing a pragmatic and utilitarian position in the popular reflection on the genetic event. The perception of risk/benefit determines rejection or acceptance (Dass, Anjum, and Gupta 2018). Differences in perceived risk between experts and civil society have been found to accentuate divergences in acceptance (Savadori et al., 2004).

Also, people’s attitudes toward technology and its products are linked to the perception of right and wrong (Dass et al., 2018). For example, communities accept GMOs in the biopharmaceutical industry and research with minimal objections. Likewise, transgenic drugs such as insulin, GH, erythropoietin, and others are generally viewed as good and receive some community approval (Rzymski and Królczyk 2016).

Kazana et al., in a pioneering study on attitudes towards transgenic forest trees, demonstrated a similarity of attitudes among European and non-European university communities. The participants showed the criterion of using transgenic trees only in controlled areas without being released into the environment. The level of knowledge on transgenic trees leaned towards the concept but not towards the current status of their local or global use. There was also support for mandatory labeling as a requirement for free choice by the population. These considerations still suggest a state of distrust towards this type of organism, which stigmatizes its presence in society (Kazana et al., 2015)

However, the perceived threat of GMOs and the perceived harm to health, the environment, or the natural order are criteria used by civil society (Scott et al., 2018). GMO foods are roundly rejected by fractions of civil society, perhaps as a manifestation of food phobia (Faccio and Nai, 2019). For example, in China, the population perception is primarily against GMO foods (Cui and Shoemaker, 2018), having political and economic causes related to structures that mediate the production process, marketing, and regulation. Similar situations are repeated in other countries such as Bosnia and Herzegovina (Bevanda et al., 2017), Tanzania (Mnaranara et al., 2017), and Mexico (Robayo et al., 2018).

In Ecuador, the attitude toward GMOs is marked by a set of social, educational, and political factors that have established a position of rejection (Paz-y-Mino et al., 2013). The conception of food sovereignty, biodiversity conservation, and environmental protection is part of the Ecuadorian cultural tradition, embodied at the constitutional level (Maluf et al., 2018). The view of GMOs as a threat to these cultural traditions is materialized in broad opposition in various political, academic, and civil circles (Bravo 2017).

Also the history of discredit in the mass media from a political perspective, the veto at the constitutional level for research and productive purposes, or the short term of its possible use under approval by presidential decree makes the population perceive threats over the benefits (Paz-y-Mino et al., 2013). Environmental campaigns propose a GMO-free Ecuador to achieve the health of the population and the conservation of the environment (Intriago and Bravo, 2015).

A relevant aspect to consider is the criterion for using GMOs under strict biosafety and biosecurity norms, revealing a reflection on the ethical assumptions that should guide the use of GMOs in Ecuador. Currently, there is no biosafety code for the use of GMOs in the country, even though its elaboration began in 2015 (Implementación del Marco Nacional de Bioseguridad, 2015).

According to the authors’ criteria, several reasons may have an impact on the attitude toward GMOs. Some of them are associated with the massive lack of knowledge and the lack of instruction about new generation biotechnologies in curriculum at the higher primary and undergraduate levels. Research in students has found rational thinking in arguing the use of genetic technology, avoiding emotional arguments (Črne-Hladnik et al., 2012). That is why the authors support the approach used in research where students are the analytic unit and can be decisive to find ethical arguments in the population. It has also been corroborated that a lack of education and knowledge can be associated with the acceptance of GMOs (Cacciatore, 2021). According to the Dunning-Kruger model, the population’s limited knowledge fosters high certainty of rejection towards GMOs. However, this fact is modified by the acquisition of more specific knowledge on the subject. There have also been minimal opportunities for debate between civil society and academic and political structures to educate and dialogue with the population about GMOs, resulting in a confrontation between science and the defenders of Pachamama (Mother Earth in Quechua language) (Paz-y-Mino et al., 2013).

Attitudes around GMOs have been associated with a set of sociodemographic variables such as religion, conceptions of life and nature, knowledge, educational level, area of academic training, geographic region, and culture (Öz et al., 2018). Modeling attitudes toward GM salmon in Malaysia using structural equations revealed the existence of a complex phenomenon with multiple explanatory variables (Amin et al., 2014). The predominant dimension was risk perception, although perceived benefits were also relevant. This fact coincides with findings in the Ecuadorian population (highly religious), where risks and benefits are perceived independently. The distorted beliefs of the Ecuadorian population on aspects related to GMOs such as biosafety and biosecurity have a direct impact on average acceptance. Also, the limited life experiences of the population with GMOs restrict practical knowledge.

The bioethical approach of the civilian population is a dimension explored in the explanation of attitudes towards GMOs. Exploring moral, utilitarian, personalist, and principal-based stances contribute to understanding the root causes of attitudes. Harfouche and collaborators showed that the ethical stance and values are decisive in the acceptance and trust of society towards GMOs as technology or their consumption as a product (Harfouche et al., 2021).

The collectivist and liberalist philosophical basis for using GMOs proposes two irreconcilable opposing extremes: greater good for the most significant number of people and individual freedom. Principlism endorses genetic modification as an ethical act proper to the autonomy of the scientist in the research. This position exalts freedom as the main good, ignoring possible consequences of scientific activity in the immediate future. The utilitarian approach arises the benefits obtained due to genetic modification of organisms for the people or where the benefit outweighs the existing risks (Appiah, 2015). This view argues for the extensive use of biotechnology to mitigate hunger in vast regions of the planet (Harfouche et al., 2021).

The anthropological personalist bioethical arguments propose humans as an end in themselves. The superiority of humans over the rest of the species justifies genetic modification, as long as the end itself is the wellbeing of humans. This anthropocentric position establishes the person over the rest of living organisms, minimizing the ecological conception of human life. However, there are more conciliatory positions with nature and living beings that integrate and respect living beings or the ecosystem as a whole. The biocentrist and ecocentrist currents have managed to reconcile humans with their environment to achieve the necessary sustainability of the ecosystem and curb environmental deterioration in this new era of the Anthropocene (Lee, 2017).

The virtue ethics proposes the acceptance of GMOs under strict *in situ* and *ex situ* regulatory measures. The application of bioethical principles such as responsibility and precaution allows for the regulation of GMO technology (Appiah, 2015). According to the author, the responsible use of GMO technology must fallows four main guidelines: search for the wellbeing of humans and their environment, future projection on possible effects, participation of all sectors of society in the approval of its use, and broad accessibility to all sectors of society.

Bremer et al., in their case study research on attitudes towards fast-growing transgenic salmon in Europe, emphasized a systemic and pluralistic reflection (Bremer et al., 2015). The participation of productive and scientific entities of private or public profile and civil society can bring together different ethical thoughts in an open dialogue between decision-makers and society. Furthermore, the use oftools such as the ethics matrix for decision-making can facilitate divergent meeting thoughts. However, the authors consider the proposal made by Bremer et al. to be reductionist because it only includes the principlism bioethical approach and ignores the rest of the trends. Technology governance should consider diverse trends to receive the most significant acceptance and the least possible uncertainty.

### What Approach Should be used in Ecuador for an Adequate Governance of GMOs?

Even though there is no research, communication, or educational strategies on implementing GMO technology at the national and local levels. The change of position of the Ecuadorian academic population will favor actions to develop genetic engineering and biotechnology. The results obtained show UCACUE as an agent of change in this process. Therefore, this focus group represents the future consumers, policy-makers, or developers of this organism. The development of seedbeds at UCACUE could be the strategy for developing GMOs at the local level. Through educational, communicational, and participatory strategies, teachers and students could reconcile scientific and ethical criteria about GMOs. This fact suggests that the academic community could manage the national level’s research, implementation, and development of GMO technology.

The authors consider that the results obtained should be interpreted with caution due to the biases caused by the use of the questionnaire as a measurement instrument in a population of university students. Response biases related to the number of participants and cognitive biases such as the Dunning-Kruger effect may be present reducing the scope of the investigation.

GMOs have shown a clash of opinions between science and the passionate defense of national sovereignty, the environment, and human health in Ecuador. Passions have marginalized scientific thoughts for the sake of preserving national culture and identity, health, the environment, and Ecuador’s good living. The effect has been to provoke attitudes of rejection and fear toward this technology from extreme positions. However, there is currently a slight change of position with a tendency toward acceptance in the academic sector, corroborating a transformation in the thinking of Ecuadorian civil society toward GMOs. Considerations on the use of GMOs are supported by an incipient bioethical stance that leans toward a pragmatic utilitarianism based on the immediate or mediate benefits of the technology.

The use of GMOs in Ecuador must contemplate a process of change in the civilian population’s perception of them. Dialogue among the productive, technological, scientific, academic, civil sectors, and the minorities and indigenous communities of society will make it possible to unify criteria and smooth differences over in this field of technology. The intervention of variables such as knowledge, the bioethical approach, beliefs, and practices with GMOs would be decisive in achieving their inclusion within the Ecuadorian science and technology system under the perspective of responsible research and innovation.

## Data Availability

The original contributions presented in the study are included in the article/Supplementary Material; further inquiries can be directed to the corresponding author.

## References

[B1] Amin L. Azad M. A. K. Gausmian M. H. Zulkifli F. (2014). Determinants of Public Attitudes to Genetically Modified Salmon. PLoS ONE. 9 (1), e86174. 10.1371/journal.pone.0086174 24489695 PMC3906022

[B2] Appiah S. K. (2015). The Debate on the Use of Genetic Technology and Production of GM Foods in Ghana: Ethical Perspectives. Cont. J. Afr. Stud. 3 (2), 113–134. 10.4314/contjas.v3i2.5

[B3] Bevanda L. Žilić M. Ećimović B. Matković V. (2017). Public Opinion toward GMOs and Biotechnology in Bosnia and Herzegovina In edited by Badnjevic A., CMBEBIH 2017 IFMBE Proceedings. Singapore: Springer. 10.1007/978-981-10-4166-2_70

[B4] Bravo E. (2017). Visiones y Tensiones Sobre el Debate de los Transgénicos en el Ecuador. Perspectivas Rurales Nueva Época 15 (30), 11–29. December. 10.15359/prne.15-30.1

[B5] Bremer S. Millar K. Wright N. Kaiser M. (2015). Responsible Techno-Innovation in Aquaculture: Employing Ethical Engagement to Explore Attitudes to GM Salmon in Northern Europe. Aquaculture. 437 (febrero), 370–381. 10.1016/j.aquaculture.2014.12.031

[B6] Cacciatore M. A. (2021). Misinformation and Public Opinion of Science and Health: Approaches, Findings, and Future Directions. Proc. Natl. Acad. Sci. USA. 118 (15), e1912437117. 10.1073/pnas.1912437117 33837143 PMC8053916

[B7] Črne-Hladnik H. Hladnik A. Javornik B. Košmelj K. Peklaj C. (2012). Is Judgement of Biotechnological Ethical Aspects Related to High School Students' Knowledge? Int. J. Sci. Education. 34 (8), 1277–1296. 10.1080/09500693.2011.572264

[B8] Cui K. Shoemaker S. P. (2018). Public Perception of Genetically-Modified (GM) Food: A Nationwide Chinese Consumer Study. Npj Sci. Food. 2 (1), 10. 10.1038/s41538-018-0018-4 31304260 PMC6550219

[B9] Dass P. Anjum N. Gupta D. (2018). Genetically Modified Organisms: Reliability Analysis and Perceptions. J. Inf. Optimization Sci. 39 (7), 1401–1415. 10.1080/02522667.2018.1441245

[B10] Faccio E. Guiotto Nai Fovino L. (2019). Food Neophobia or Distrust of Novelties? Exploring Consumers' Attitudes toward GMOs, Insects and Cultured Meat. Appl. Sci. 9 (20), 4440. 10.3390/app9204440

[B11] Gatica-Arias A. (2020). The Regulatory Current Status of Plant Breeding Technologies in Some Latin American and the Caribbean Countries. Plant Cell Tiss Organ Cult. 141 (2), 229–242. 10.1007/s11240-020-01799-1

[B12] Gbashi S. Adebo O. Adebiyi J. A. Targuma S. Tebele S. Areo O. M. (2021). Food Safety, Food Security and Genetically Modified Organisms in Africa: a Current Perspective. Biotechnol. Genet. Eng. Rev. 37 (1), 30–63. 10.1080/02648725.2021.1940735 34309495

[B13] Gudynas E. (2009). La Ecología Política del Giro Biocéntrico en la Nueva Constitución de Ecuador. Rev.estud.soc. 32 (June), 34–47. 10.7440/res32.2009.02

[B14] Harfouche A. L. Petousi V. Meilan R. Sweet J. Twardowski T. Altman A. (2021). Promoting Ethically Responsible Use of Agricultural Biotechnology. Trends Plant Sci. 26 (6), 546–559. 10.1016/j.tplants.2020.12.015 33483266

[B15] Implementación del Marco Nacional de Bioseguridad (2015). Proyecto. Ecuador. Available at: http://www.ambiente.gob.ec/wp-content/uploads/downloads/2015/08/BIOSEGURIDAD.pdf .

[B16] Intriago Barreno R. S. Bravo Velásquez E. (2015). Situación Actual del Ecuador Como Territorio Libre de Transgénicos. Lv. 18, 264–226. 10.17141/letrasverdes.18.2015.1606

[B34] Kazana V. L. Tsourgiannis V. Iakovoglou C. Stamatiou A. Alexandrov S. Araújo S. (2015). Public Attitudes towards the Use of Transgenic Forest Trees: A Cross-Country Pilot Survey. IForest - Biogeosciences and Forestry 9 (2), 344. 10.3832/ifor1441-008

[B17] Lee L. M. (2017). A Bridge Back to the Future: Public Health Ethics, Bioethics, and Environmental Ethics. Am. J. Bioeth. 17 (9), 5–12. 10.1080/15265161.2017.1353164 28829266

[B18] Maluf F. Calaça I. Freitas P. Augusto S. (2018). La Naturaleza Como Sujeto de Derechos: Análisis Bioético de las Constituciones de Ecuador y Bolivia. Rev. Latinoam. Bioet. 18 (34–1), 155–171. 10.18359/rlbi.3030

[B19] Manoj K. Ratwan P. (2018). Transgenic or Genetically Modified Farm Animals and Their Applications: a Review. Res. Rev. J. Vet. Sci. Technology. 5 (3), 25–34. 10.37591/rrjovst.v5i3.560

[B20] Martin H. M. Durr D. Smith L. M. Finke R. Cherry A. (2017). Analysis of GMO Food Products Companies: Financial Risks and Opportunities in the Global Agriculture Industry. Afr. J. Econ. Sustainable Development. 6 (1), 1–17. 10.1504/AJESD.2017.082813

[B21] Mnaranara T. E. Zhang J. Wang G. (2017). Public Perception Towards Genetically Modified Foods in Tanzania. J. Anim. Plant Sci. 27 (2), 589–602. Available at: http://www.thejaps.org.pk/docs/v-27-2/30.pdf .

[B22] Oleas M. Tejada E. Lascano R. (2016). Conocimientos y Aceptación de Alimentos Transgénicos en Adolescentes de la Provincia de Imbabura, Ecuador. Rev. Esp Nutr. Comunitaria. 26 (1), 9. Available at: http://www.renc.es/imagenes/auxiliar/files/RENC_2016_1-02._Oleas_M_Conocimientos_transgenicos_Imbabura(1).pdf .

[B23] Öz B. Unsal F. Movassaghi H. (2018). Consumer Attitudes Toward Genetically Modified Food in the United States: Are Millennials Different? J. Transnational Management. 23 (1), 3–21. 10.1080/15475778.2017.1373316

[B24] Pardo R. Midden C. Miller J. D. (2002). Attitudes Toward Biotechnology in the European Union. J. Biotechnol. 98 (1), 9–24. 10.1016/s0168-1656(02)00082-2 12126802

[B25] Paull J. Hennig B. (2019). New World Map of Genetically Modified Organism (GMO) Agriculture: North and South America Acres Australia (January) 101, 59–60. Available at: https://www.researchgate.net/profile/John-Paull/publication/336411623_New_World_Map_of_Genetically_Modified_Organism_GMO_Agriculture_North_and_South_America_85/links/5da01b7ba6fdcc8fc346da67/New-World-Map-of-Genetically-Modified-Organism-GMO-Agriculture-North-and-South-America-85.pdf .

[B26] Paz-y-Mino C. Binsfeld P. Torres M. Arahana V. Bravo E. Fornasini M. (2013). Transgénicos: Una Cuestión Científica. 1ra ed. Quito. UDLA. Available at: https://www.researchgate.net/profile/Cesar-Paz-Y-Mino/publication/326504306_Transgenicos_Una_cuestion_cientifica/links/5b8817f3a6fdcc5f8b71fde6/Transgenicos-Una-cuestion-cientifica.pdf .

[B27] Robayo A. Galindo M. Yáñez L. Aldama C. (2018). Measurement of Public Perception of GMOs With a Likert-type Scale. Agrociencia (Montecillo). 52 (5), 767–781. Available at: https://www.cabdirect.org/cabdirect/abstract/20203084608 .

[B28] Robinson AW T. Rajakaruna A. (2016). Application of Recombinant DNA Technology (Genetically Modified Organisms) to the Advancement of Agriculture, Medicine, Bioremediation and Biotechnology Industries. J. Appl. Biotechnol. Bioeng. 1 (3), 78. 10.15406/jabb.2016.01.00013

[B29] Rzymski P. Królczyk A. (2016). Attitudes Toward Genetically Modified Organisms in Poland: To GMO or Not to GMO? Food Sec. 8 (3), 689–697. 10.1007/s12571-016-0572-z

[B30] Savadori L. Savio S. Nicotra E. Rumiati R. Finucane M. Slovic P. (2004). Expert and Public Perception of Risk From Biotechnology. Risk Anal. 24 (5), 1289–1299. 10.1111/j.0272-4332.2004.00526.x 15563295

[B31] Scott S. E. Inbar Y. Wirz C. D. Brossard D. Rozin P. (2018). An Overview of Attitudes Toward Genetically Engineered Food. Annu. Rev. Nutr. 38 (1), 459–479. 10.1146/annurev-nutr-071715-051223 29801421

[B32] Smyth S. J. Kerr W. A. Phillips P. W. B. (2015). Global Economic, Environmental and Health Benefits from GM Crop Adoption. Glob. Food Security. 7 (diciembre), 24–29. 10.1016/j.gfs.2015.10.002

[B33] Trigo E. Traxler G. Pray C. Echeverría R. (2002). Biotecnología agrícola y desarrollo rural en América Latina y el Caribe. RUR-107. Serie de informes técnicos del Departamento de Desarrollo Sostenible. Whashington DC: Banco Interamericano de Desarrollo. Available at: https://d1wqtxts1xzle7.cloudfront.net/47904405/getdocument-with-cover-page-v2.pdf?Expires=1630594858&Signature=OEyXxug6YrbEvMhzolqyEs3bm5e∼flAZQ9QGRuk6vP2qwpGQlQHEKtbIKNQthhvEe1gmNKlJXtLZEWjkXlg7∼MlzVguhPP78EJhtJkkKzLe3o1oiZ6gm0TbKRJGe76rscg1Xmk-F0f2fqcrlKFA∼PhBecneUsGqInsjTxZsZfhd∼xkDYlJh-TrZGtvAZmPGOeN5JzusVrsiEav8vU4-w2svZKvLZNGB8G0pFJzIWQ7L1ODSuVgUZxrRS2DzOWrm3hPRHzM2eyC1krI3e1u2a4nfWwQSLZ4gHQ8gxOXdK∼z9PAro5kLbXfOZL-mZWGvIFxKpDqdmMbmCs3Oz3fZlUyw__&Key-Pair-Id=APKAJLOHF5GGSLRBV4ZA .

