# Supplementary material for: Attitudes of the Ecuadorian University Community Toward Genetically Modified Organisms
Source: Front Bioeng Biotechnol. 2022 Feb 18;9:801891. doi: 10.3389/fbioe.2021.801891 (PMC8894883; doi:10.3389/fbioe.2021.801891)
Supplement: Supplementary file 1 [file DataSheet4.docx]

Figure 1- Sociodemographic characteristics of the UCACUE community, March- August 2020, Ecuador.
