# Supplementary material for: Attitudes of the Ecuadorian University Community Toward Genetically Modified Organisms
Source: Front Bioeng Biotechnol. 2022 Feb 18;9:801891. doi: 10.3389/fbioe.2021.801891 (PMC8894883; doi:10.3389/fbioe.2021.801891)
Supplement: Supplementary file 2 [file DataSheet5.docx]

| Table 3- Summary measures of the linear regression model ^a,b^.. | | | | | | | | | | | | | | | | | | |  |
| --- | --- | --- | --- | --- | --- | --- | --- | --- | --- | --- | --- | --- | --- | --- | --- | --- | --- | --- | --- |
| R | | R^2^ | Adjusted R^2^ | Standard error R^2^ | | Change statistics | | | | | | | | | Durbin-Watson | | ANOVA | |  |
|  |  |  |  |  |  | R^2^ Change | | F Change | | df1 | df2 | Sig. F Change | | |  |  |  |  |  |
| ,807 | | ,652 | ,647 | ,600 | | ,652 | | 149,444 | | 10 | 719 | ,000 | | | 1,951 | | F=149,44  p=,000 | |  |
|  | |  |  |  | |  | |  | |  |  |  | | |  | |  | |  |
| Model | | | | | Unstandardized coefficients | | | | Standardized coefficients | | | t | Sig. | 95,0 % Confidence Interval B | | | | | |
|  |  |  |  |  | B | | Standard error | | Beta | | |  |  | Lower limit | | Upper limit | | | |
|  | (Constant) | | | | -,697 | | ,165 | |  | | | -4,235 | ,000 | -1,021 | | -,374 | | | |
|  | Sex | | | | ,078 | | ,046 | | ,039 | | | 1,709 | ,088 | -,012 | | ,168 | | | |
|  | Place of residence | | | | ,069 | | ,061 | | ,025 | | | 1,118 | ,264 | -,052 | | ,189 | | | |
|  | GMOs beliefs | | | | ,432 | | ,043 | | ,300 | | | 9,998 | ,000 | ,347 | | ,516 | | | |
|  | Practices with GMOs | | | | ,147 | | ,033 | | ,117 | | | 4,507 | ,000 | ,083 | | ,211 | | | |
|  | GMOs Knowledge | | | | ,050 | | ,014 | | ,082 | | | 3,460 | ,001 | ,021 | | ,078 | | | |
|  | Bioethical approach towards GMO | | | | ,106 | | ,006 | | ,495 | | | 16,867 | ,000 | ,094 | | ,118 | | | |
|  | Religión | | | | ,020 | | ,014 | | ,033 | | | 1,472 | ,141 | -,007 | | ,048 | | | |
|  | Food expenditures (USD) | | | | -,047 | | ,038 | | -,032 | | | -1,217 | ,224 | -,122 | | ,029 | | | |
|  | Household income (USD) | | | | ,051 | | ,033 | | ,042 | | | 1,567 | ,118 | -,013 | | ,115 | | | |
|  | Educational level | | | | ,005 | | ,038 | | ,004 | | | ,136 | ,892 | -,069 | | ,080 | | | |
|  | Academic training | | | | ,011 | | ,016 | | ,015 | | | ,663 | ,508 | -,21 | | ,42 | | | |
|  |  | | | |  | |  | |  | | |  |  |  | |  | | | |
| 1. Predictors: (Constant), Sex, Place of residence, GMOs beliefs, Practices with GMOs, GMOs Knowledge, Bioethical approach towards GMO, Religion, Food expenditures (USD), Household income (USD), Educational level, Academic training. | | | | | | | | | | | | | | | | | |  |  |
| b. Dependent variable: Attitude towards GMOs | | | | | | | | | | | | | | | | | |  |  |
