# Supplementary material for: Attitudes of the Ecuadorian University Community Toward Genetically Modified Organisms
Source: Front Bioeng Biotechnol. 2022 Feb 18;9:801891. doi: 10.3389/fbioe.2021.801891 (PMC8894883; doi:10.3389/fbioe.2021.801891)
Supplement: Supplementary file 4 [file DataSheet1.PDF]

Universidad Católica de Cuenca

# CUESTIONARIO

## ACTITUDES HACIA LOS OGM

El cuestionario es parte de una investigación de la UCACUE con vistas a caracterizar creencias, prácticas y conocimiento sobre los **Organismos Genéticamente Modificados (OGM) o Transgénicos** en la población de Ecuador. Su participación es anónima, voluntaria y decisiva en dicho proceso.

**INSTRUCCIONES**

- Por favor lea cada pregunta y responda de manera espontánea, marcando la alternativa correspondiente.
- No hay respuestas buenas ni malas, correctas o incorrectas.
- Evite interrupciones y hablar con sus compañeros mientras conteste las preguntas; así garantiza una máxima concentración y fiabilidad. Si considera marcar una nueva respuesta, hágalo anulando la respuesta anterior.

## 1. DATOS SOCIODEMOGRÁFICOS

**Edad** ____ **Sexo:** F___ M___ **Lugar de residencia:** Urbano____ Rural____

**Nivel educativo:** Ninguno___ Básico___ Básico Superior___ Universitario___ MSc, PhD___

**El ingreso económico familiar en USD es:**

Menor a 800 ___ Entre 801 y 1600 __ Mayor a 1600___

**Ocupación actual:** Estudiante___ Docente___ Trabajador ___ Comerciante____ Desempleado___

**Alimento de mayor consumo (marque uno):**

Golosinas ____ Procesados____ Enlatados y embutidos____ Naturales ____

**Gastos en alimentación por mes:** Menos de 200 usd____ Entre 200 y 400 usd___ Más de 400 usd___

**Área del conocimiento donde trabaja:**

Ninguna___ Salud___ Ciencias Sociales___ Bioética___ Ingenierías___ Ciencias Exactas___

**Área formación académica en pregrado y posgrado:**

Ninguna___ Salud___ Ciencias Sociales___ Bioética___ Ingenierías___ Ciencias Exactas___

**Religión de práctica:** Católica___ Protestante___ Musulmana___ Afroecuatoriana___ Ninguna ___

**Tengo preocupaciones por el cuidado del medio ambiente ( Entre 1 Nada --- 5 Mucho):** _____

**Tiene ud algún conocimiento sobre los OGM (Transgénicos)** Si____ No ____

**Si respondió NO de por terminado este cuestionario.**

## I- CREENCIAS SOBRE ORGANISMOS GENETICAMENTE MODIFICADOS (OGM)

| Manifieste **el grado de concordancia de su postura** para cada afirmación que se presenta, **colocando el número correspondiente** en un grado de:<br>**1(Totalmente en Desacuerdo) ------------------------------ hasta 5 (Totalmente de Acuerdo)** | **Grado de concordancia 1,2,3,4,5** |
|---|---|
| a) Los OGM son mal llamados organismos transgénicos. | |
| b) Los OGM pueden ser usados como alimentos para el hombre y los animales | |
| c) Los OGM son usados en la producción de medicinas para el hombre y los animales. | |
| d) Los OGM se utilizan como modelos en la investigación científica. | |
| e) Los OGM son una tecnología beneficiosa para la humanidad. | |
| f) Los OGM contribuyen con el desarrollo científico del mundo. | |
| g) Los OGM son inócuos (no dañinos) para la salud del ser humano. | |
| h) La tecnología de OGM es generalmente segura para el planeta y los ecosistemas. | |
| i) La tecnología de OGM tiene grandes ventajas económicas para los países que la aplican | |
| j) La tecnología de OGM es favorecida por las leyes nacionales. | |
| k) La tecnología de OGM debe estar regulada bajo estrictas normas de bioseguridad, amparadas bajo la ley nacional y supranacional. | |
| l) Los OGM deben estar confinados (aislados) bajo medidas de bioseguridad estrictas. | |
| m) La decisión de producción y uso de los OGM debe ser tomada por los científicos del país, conjuntamente con la sociedad. | |
| n) La tecnología de OGM es factible para los países en vías de desarrollo como Ecuador. | |
| o) La información que existe sobre los OGM es clara y precisa. | |
| p) La tecnología de OGM debe ser usada con precaución por parte la comunidad científica | |

| II- PRÁCTICAS SOBRE ORGANISMOS GENETICAMENTE MODIFICADOS (OGM) Escala (6-30) | |
|---|---|
| Manifieste el **grado de concordancia** de su postura para cada afirmación que se presenta, colocando el número correspondiente en un grado de:<br>**1(Totalmente en Desacuerdo) --------------------------------hasta 5 (Totalmente de Acuerdo)** | **Grado de concordancia 1,2,3,4,5** |
| a) En la dieta incluyo OGM como alimentos (aceite soya, snacks, embutidos, otros) | |
| b) Utilizo OGM durante mi desempeño laboral o estudiantil. | |
| c) Busco información sobre OGM en diferentes fuentes (internet, revistas, colegas de trabajo y/o estudio). | |
| d) He sugerido a otros colegas, conocidos o familiares que utilicen los OGM como alimentos o fuente de medicamentos. | |
| e) He participado en campañas o debates que tratan el tema de OGM. | |
| f) Reflexiono sobre los OGM y su impacto en la vida del hombre y la naturaleza. | |

| III- ACTITUDES HACIA LOS ORGANISMOS GENETICAMENTE MODIFICADOS (OGM) (Escala 6-30) | |
|---|---|
| Manifieste el **grado de concordancia** de su postura para cada afirmación que se presenta, colocando el número correspondiente en un grado de:<br>**1(Totalmente en Desacuerdo) ------------------------------- hasta 5 (Totalmente de Acuerdo)** | **Grado de concordancia 1,2,3,4,5** |
| a) Apruebo el uso de la tecnología de OGM en el país bajo estrictas normas de bioseguridad. | |
| b) Estoy a favor del uso de los OGM en la investigación científica | |
| c) Apruebo los OGM para la alimentación humana | |
| d) Apruebo los OGM para la alimentación de animales de cría | |
| e) Apruebo los OGM con vistas a producir medicamentos para el hombre y los animales | |
| f) Apruebo el uso de los OGM para el cuidado del medio ambiente | |

## IV- Nivel de Conocimiento sobre OGM

Manifieste SU conocimiento sobre ORGANISMOS GENETICAMENTE MODIFICADOS (OGM) encerrando en un círculo la respuesta que Usted considere correcta para cada afirmación que se presenta. Seleccione una opción.

**1 Un organismo genéticamente modificado (OGM) es:**

a) Organismo con genotipo modificado obtenido mediante cruzamiento natural entre una misma especie
b) Organismo con genotipo modificado por tecnología de ADN recombinante en un laboratorio.
c) Organismo con genotipo modificado mediante técnicas de mejoramiento genético
d) No se

**3. Los OGM son de naturaleza:**

a) Animal y Vegetal
b) Animal y Microorganismos
c) Microorganismos y Vegetales
d) Vegetal, Animal, Microorganismos
e) No se

**4. Un OGM empleado como modelo en la investigación científica es:**

a) Ratón
b) Maíz
c) Salmón
d) Cerdo
e) No se

**5. Una de las aplicaciones principales de los OGM es la obtención de medicamentos como:**

a) antibióticos
b) insulina
c) aceite de soja
d) vitaminas
e) No se

**6. Entre los países latinoamericanos que han fortalecido la industria farmacéutica por la tecnología de OGM está:**

a) Ecuador
b) Venezuela
c) Bolivia
d) Cuba
e) No se

**7. La tecnología principal para la obtención de OGM es:**

a) Biotecnología
b) Ingeniería Genética
c) Ingeniería de Alimentos
d) Mejoramiento genético
e) No se

**8. En América Latina la obtención de OGM está prohibida a nivel de la Constitución en:**

a) Ecuador
b) Argentina
c) Cuba
d) Chile
e) No se

**9. Un acuerdo supranacional sobre el uso de los OGM es:**

**a)** Cumbre de Río
**b)** Convenio de Cartagena
**c)** Protocolo de Kyoto
**d)** Cumbre del Medio Ambiente
**e)** No se

**10. Un OGM utilizado como alimento para el ser humano es:**

a) arroz
b) alfalfa
c) maíz
d) algodón
e) No se

| V- POSICIÓN BIOÉTICA ACERCA DE ORGANISMOS GENETICAMENTE MODIFICADOS | |
|---|---|
| Manifieste el **grado de concordancia** de su postura para cada afirmación que se presenta, colocando el número correspondiente en un grado de: **1(Totalmente en Desacuerdo) -------------------------------- hasta 5 (Totalmente de Acuerdo)** | **Grado de concordancia 1,2,3,4,5** |
| a) El uso de la tecnología de los OGM es un derecho de los investigadores y la sociedad de ejercer su autonomía científica. | |
| b) El uso de la tecnología de OGM debe ser permitido porque tiene más beneficios que perjuicios para la sociedad y el ser humano. | |
| c) La tecnología de OGM puede ser utilizada por el hombre pues es un ente superior que tiene el poder de modificar a otros organismos según su consideración y criterio. | |
| d) La tecnología de OGM es válida porque intenta mejorar una forma de vida respetando su integridad física. | |
| e) El uso de la tecnología de OGM se justifica solo si es practicada por científicos virtuosos que busquen el bien y lo bueno para la humanidad. | |

**CONSENTIMIENTO DE PARTICPACIÓN EN ESTUDIO DE INVESTIGACIÓN**

**Institución:** Universidad Católica de Cuenca

**Investigador:** Carlos A Román Collazo

email: cromanc@ucaue.edu.ec; celular:0987965030

**Título:** ACTITUDES SOBRE ORGANISMOS GENETICAMENTE MODIFICADOS EN ECUADOR, MARZO – AGOSTO 2020.

**Propósito del estudio:** Identificar el nivel de conocimiento, prácticas y actitudes sobre los transgénicos en los estudiante y docentes de la UCACUE.

**Procedimiento:** Se aplicará a los estudiantes y docentes un cuestionario auto administrado con una duración aproximada de 15 minutos en el espacio docente universitario, via online.

Se les explicará a los estudiantes y docentes que la participación es voluntaria, pudiendo desistir de responder en cualquier momento sin perjuicio alguno.

**Riesgos:** No existe riesgo para los participantes de este estudio.

**Beneficios:** No existe beneficio económico por participar en el estudio.

**Costo e incentivo:** El estudio es totalmente gratuito.

**Confidencialidad:** La información proporcionada será absolutamente confidencial y anónima. La publicación de los resultados será comunicada a los participantes sin mostrar información que permita la identificación de las personas. Los archivos serán de domino exclusivo de los investigadores.

**Autorización:** Mediante la presente firma doy el consentimiento informado para la participación en este estudio.
