# Supplementary material for: Attitudes of the Ecuadorian University Community Toward Genetically Modified Organisms
Source: Front Bioeng Biotechnol. 2022 Feb 18;9:801891. doi: 10.3389/fbioe.2021.801891 (PMC8894883; doi:10.3389/fbioe.2021.801891)
Supplement: Supplementary file 5 [file DataSheet2.docx]

| Table 2- Items that make up the Attitude towards GMOs dimension. | | | |
| --- | --- | --- | --- |
| Items that makeup Attitude towards GMOs Dimension | Mean | Standard deviation | Mean´s comparison (Wilcoxson´s Test) |
| 1. I approve of the use of GMOs technology in the country under strict biosafety regulations. | 3,17 | 1,21 | B > A  Z= -5,638  p=0,000  A = F  Z= -0,309  p=0,758  A > C  Z= -10,822  p=0,000  C = D  Z= -0,570  p=0,568 |
| 1. I am in favor of the use of GMOs in scientific research | 3,39 | 1,20 |  |
| 1. I approve GMOs for human consumption. | 2,72 | 1,17 |  |
| 1. I approve GMOs for feeding farmed animals | 2,71 | 1,18 |  |
| 1. I approve GMOs to produce medicines for humans and animals. | 3,06 | 1,18 |  |
| 1. I approve the use of GMOs for the care of the environment | 3,16 | 1,26 |  |
