# Supplementary material for: Attitudes of the Ecuadorian University Community Toward Genetically Modified Organisms
Source: Front Bioeng Biotechnol. 2022 Feb 18;9:801891. doi: 10.3389/fbioe.2021.801891 (PMC8894883; doi:10.3389/fbioe.2021.801891)
Supplement: Supplementary file 6 [file DataSheet1.docx]

| Table 1- Descriptive statistic of the Attitude, Beliefs, Practices and Knowledge on GMOs variables in UCACUE, 2020. | | | | |
| --- | --- | --- | --- | --- |
| Variable | X +- SD | CI 95% | Min-Max | Instrument scale |
| Attitude towards GMO | 3,03+-1,01 | 2,96-3,11 | 1-5 | 1-5 |
| Beliefs about GMO | 3,33 +-0,70 | 3,28-3,38 | 1-5 | 1-5 |
| Practices with GMO | 2,54+-0,80 | 2,48-2,60 | 1-5 | 1-5 |
| Knowledge about OGM | 1,95+-1,66 | 1,81-2,05 | 0-8 | 0-9 |
| Bioethic approach | 2,98 +-0,94 | 2,92-3,06 | 1-5 | 1-5 |
| X+-SD- mean +- standard deviation  CI- Confidence interval  Min- Minimun  Max- Maximum | | | | |
